# Realist-informed review of motivational interviewing for adolescent health behaviors

**DOI:** 10.1186/s13643-018-0767-9

**Published:** 2018-07-27

**Authors:** Christina Mutschler, Erica Naccarato, Jen Rouse, Caitlin Davey, Kelly McShane

**Affiliations:** 1 Ryerson University, Toronto, Canada; 2 University of Waterloo, Waterloo, Canada

**Keywords:** Motivational interviewing, Health behaviors, Adolescent, Realist review

## Abstract

**Background:**

Clinical research investigating effective intervention strategies for adolescents to improve health behaviors has shifted to the application of motivational interviewing (MI). Evidence indicates that MI is an effective intervention for improving health behaviors as related to diet, exercise, and diabetes among adolescents. However, there is a lack of understanding about the mechanisms through which MI works and the contextual factors impacting MI effectiveness. The purpose of this review was to understand how, for whom, and under what circumstances MI works for adolescent health behavior change, which will inform future implementation of this intervention. To provide this in-depth understanding, a realist-informed systematic review was conducted in order to synthesize the evidence on the use of MI for health behaviors. Self-determination theory (SDT) was chosen as the candidate theory for testing in the present review.

**Methods:**

Databases including PsycINFO, Healthstar, Cochrane, and PubMed were searched for articles published until March 2017. The search strategy included studies that examined or reviewed the effectiveness or efficacy of MI to change health behaviors among adolescent populations. The search identified 185 abstracts, of which 28 were included in the review. The literature was synthesized qualitatively (immersion/crystallization) and tested SDT as the candidate theory.

**Results:**

Based on SDT, three mechanisms were found within reviewed studies, including competence, relatedness, and autonomy. The following contexts were found to impact mechanisms: school setting, clinician MI proficiency, parental involvement, and peer involvement.

**Conclusions:**

This realist-informed systematic review provides advances in understanding the mechanisms involved in MI for adolescent health behavior change. Additionally, it provides important practical information as to which contexts create the conditions for these mechanisms to occur, leading to health behavior change. The results can inform future MI interventions for adolescent health behavior change. Future research should continue to test this realist theory and also examine mechanism variables not extensively documented in order to improve our understanding of MI in this population.

## Background

In recent decades, the prevalence of childhood obesity in Canada and the USA has risen dramatically, with some studies estimating that as many as one in three children are overweight or obese [1–6]. Paralleling the rise of childhood obesity rates is a tenfold rise in type 2 diabetes among children and youth [7, 8]. Diabetes and obesity are associated with serious health complications, some of which can be fatal, including asthma, high blood pressure, high cholesterol, sleep apnea, joint problems, osteoarthritis, cardiovascular disease, and some cancers [1, 9, 10]. A number of risk factors for diabetes and obesity have been identified, including health behaviors such as poor diet, sedentary behavior, screen time (i.e., television and computer use), and lack of exercise [11, 12].

Clinical research investigating effective intervention strategies for adolescents to improve health behaviors is somewhat limited [12]. Recent attention has shifted to the application of motivational interviewing (MI) to target problematic health behaviors among teens with chronic health conditions [12, 13]. Evidence indicates that MI is an effective intervention for improving healthy behaviors as related to diet, exercise, and diabetes among adults [13–16]. Two recent reviews have also indicated that MI is also effective for the treatment of adolescent health behaviors [17, 18], but a gap remains as to how, why, and in what contexts MI works for adolescents, since the processes of change are somewhat unclear [19]. This understanding will provide invaluable information on future implementation of MI in this population. To provide this in-depth understanding, a realist-informed systematic review was conducted to synthesize the evidence on the use of MI for health behaviors, specifically, nutrition, eating habits, sedentary behaviors, and exercise in adolescents.

### Motivational interviewing

Motivational interviewing (MI) is a client-centered, collaborative approach that explores inconsistencies between clients’ beliefs and behaviors in order to elicit intrinsic motivation, reduce ambivalence, and evoke natural behavioral change [20]. Though a directed therapy, MI is respectful of individuals’ autonomy and ability to make decisions for themselves, and accordingly, maladaptive beliefs or cognitions are not confronted [20–22]. Therapists rely on an empathetic, empowering, and encouraging approach [12, 21, 23], where reflective listening, positive regard, and other Rogerian skills are used to support clients’ sense of self-efficacy and reinforce the therapeutic relationship, which is considered an essential element for MI effectiveness [13, 20, 24]. Self-efficacy, or the confidence that one can achieve their goals, is considered a reliable outcome predictor and is a key factor in a client’s readiness to change [24, 25].

Readiness to change is defined as a process or state of moving between no intention of making a behavior change to committing to and maintaining behavior change [23, 26]. Based on the Transtheoretical Model (TTM), individuals are at various points along this continuum of readiness to change [27–29]. Theoretically, individuals move through the stages sequentially; however, in practice, the stages are not mutually exclusive and individuals often move back and forth between stages or move through more than one stage simultaneously [22, 30]. The TTM has been criticized regarding the notion that change is predicated on the notion of stable stages or plans [31], the suggested discrete nature of the stages of change [32, 33], and the assumption of conscious decision-making and the exclusion of the role of rewards and punishment in the development of habits [34].

Research using MI to target problem health behaviors has focused on diet, nutrition, exercise, oral health, sedentary behavior, screen time, and monitoring of glucose levels [11, 15, 35]. Several reviews and meta-analyses have examined the effectiveness of MI for health behaviors and found that it is an effective intervention for diet, exercise, obesity, and diabetes among adults [12, 13–16, 31]. More specifically, MI is associated with changes in body mass index (BMI), systolic blood pressure, and total body cholesterol [16].

### Adolescent health behaviors

Adolescence is characterized as a time of significant physical, personal, and social development [36], coupled with a shift from relying heavily on parents to make decisions to engaging in more independent decision-making [19]. Health behaviors acquired in adolescence are likely to be maintained into adulthood [9, 36–38], making adolescents more responsible for their own health and lifestyle [19]. This newfound freedom may lead teenagers to eat foods low in nutrition, high in fat and/or “empty calories”; exercise well below the daily recommended amount [36, 39]; and poorly manage diabetes [40]. Thus, adolescence is a critical period for introducing interventions to halt problematic behaviors and reduce the risk of poor health outcomes or chronic disease in adulthood [41].

Research in the area of health behavior interventions for adolescents, specifically those related to obesity and diabetes, is relatively new [41]. The current literature is heavily dominated by “risky” health behaviors such as alcohol consumption, illicit drug use, cigarette smoking, and unsafe sex [12, 15]. Yet, some are beginning to add disordered eating and sedentary behavior into the fold as obesity and diabetes continue to rise in this population [39, 42–44]. MI is one intervention gaining attention in this area, though much is still unknown regarding its efficacy and the mechanisms and contexts by which it works [12, 17, 45]. Initial evidence for the effectiveness of MI for childhood and adolescent obesity and diabetes is promising [12, 46].

Two recent meta-analyses have been conducted in the area of MI for adolescent health behavior change [17, 18]. Cushing and colleagues [17] found 15 studies from their systematic search, which yielded a small but significant effect size favoring MI interventions, *g* = 0.16 (95% CI [0.05, 0.27]). The authors note the importance of reporting the training received by clinicians, in order to assess whether fidelity to MI impacts adolescent health outcomes, as the majority of studies did not report training. Additionally, they recommend that future studies analyze the effects of interventionist training on outcomes.

Gayes and Steeles [18] conducted a meta-analysis for the efficacy of MI on adolescent health behaviors, delivered to the adolescent, a parent, or both. The authors synthesized 37 articles on this topic that targeted eight health conditions including obesity, HIV/AIDS, asthma, diabetes, infant health, dental health, accident prevention, sleep, and calcium intake. The analysis found a small overall effect size for MI when compared to other active treatments and no treatment, *g* = 0.282 (95% CI [0.242, 0.323]). Interestingly, this meta-analysis also analyzed moderator variables and found that interventions delivered by community health workers yielded the largest effect size, followed by health professionals, and lastly interventionists with master’s and doctorate degrees resulted in the lowest effect size. Additionally, studies that conducted MI with the adolescent and parent together yielded significantly larger effect sizes than MI delivered with either parent or child individually. These moderator variables suggest that specific contexts in which MI is delivered to adolescents give rise to different outcomes. Therefore, further investigation into the contexts by which MI works, and how it works within these contexts, is warranted.

### Realist-informed approach to systematic reviews

Traditional systematic reviews focus on synthesizing literature and identifying whether or not interventions work. A realist approach goes beyond identifying the intervention’s efficacy to examine the underlying mechanisms and contexts in which the interventions work [45, 47]. Such an approach is predicated on the notion that an outcome occurs as a result of some events (or intervention), but there is a need to understand the generative mechanism that brings about the outcome in a particular context. These “mechanisms of change” are sometimes considered intervention or program theories that explain *how and why* outcomes occur. A realist approach states that contextual variables create the conditions by which generative mechanisms and outcomes can occur, which is explained through the construction of context-mechanism-outcome (CMO) configurations [48].

There are many differences between a realist review and a traditional systematic review, one of which includes its purpose. A realist review’s purpose is often exploratory and does not aim to determine whether an intervention works, as does a traditional systematic review. Rather a realist review aims to explore the process and contextual factors that play a role in the effectiveness or efficacy of an intervention [48]. Because of this, a realist review does not provide concrete and absolute answers and, as a result, the possibility for additional contributions to each theory may remain, as findings from a realist review are often provisional [48]. It should be noted, however, that provisional results and conclusions are common when examining mechanisms, since a sequence of studies must be completed in order to confirm the existence of particular mechanisms [49]. The present review was limited due to the small number of studies completed on the use of MI for adolescent health behaviors. Therefore, the present paper was adapted as a realist-informed review, due to the exploratory nature and, instead, is looking to develop a preliminary theory that includes some mechanism and contextual factors based on SDT. The provisional results of the realist review are often in the form of a conceptual model, which is the objective of the present realist-informed review.

### Current review and candidate theory

Recent reviews have found that MI for adolescent health behavior change is an effective intervention; however, research has yet to uncover underlying mechanisms or examine how, why, and in what contexts MI works for addressing adolescent health behaviors [12, 21]. To address such process-related issues, this review synthesized the current literature and identified outcomes, contexts, and mechanisms related to the successful application of MI to target diet, exercise, and sedentary behaviors among adolescents. Our research questions included the following: What are the mechanisms or theories by which outcomes are achieved? How does the context of the MI intervention affect mechanisms and outcomes? In order to answer these questions, we tested self-determination theory (SDT) as the candidate theory.

Recent developments in research on MI have suggested that self-determination theory (SDT) provides a useful theoretical framework for understanding the efficacy of MI [50, 51]. SDT is a theory of personality development and behavior change originating from Deci and Ryan [52], which assumes that individuals innately strive towards personal growth. The theory stipulates that autonomous behaviors are more stable and have a more positive effect on the individual [53]. SDT posits that three conditions are necessary in order to achieve autonomous behavior change [54]. The first condition is the need for competence, defined as confidence in one’s abilities and that one’s abilities can affect outcomes. Competence has been compared to other constructs, such as the development of confidence, self-efficacy, and control [55]. The second condition is the need to feel autonomous over one’s behaviors and actions, rather than feeling controlled by external sources. Autonomy is achieved when an individual feels like they have choice or ownership over their actions [55]. Third is the need to feel relatedness, which involves experiencing a connection with others and having satisfying relationships. If individuals feel a sense of caring and commitment from important people in their life, they will be more willing to make a behavior change [55]. SDT suggests that all three of these conditions can be met if the individual is in an environment that allows for self-determination to foster [54]. If an individual is in an environment where they are being controlled, it is unlikely they will develop competence, autonomy, and relatedness.

The theory states that if the social environment provides structure, autonomy support, and involvement of others, then individuals will be able to achieve the three conditions of self-determination [56]. Structure facilitates competence; autonomy support facilitates the development of autonomy; and lastly, involvement, or the perception that others care about your well-being, facilitates feelings of relatedness [53]. Therefore, the present study adopts SDT as the candidate theory for MI for adolescent health behaviors. Specifically, the intervention would be hypothesized to be most effective in contexts that allow for structured setting, that permit autonomy support, and include involvement of others. These contexts will produce the hypothesized mechanisms, which include competence, relatedness, and autonomy. These mechanisms will result in the final outcomes of health behavior change.

## Methods

### Search strategy

A literature search was conducted for all eligible published studies from 1989 up to March 2017, with the earliest paper found from 1989. This systematic review included studies that examined or reviewed the effectiveness or efficacy of MI to change health behaviors among adolescent populations. Studies that combined MI with another intervention were included as well, as long as MI was a specific focus of the study. Health behaviors included in the systematic review were related to diet and exercise. Behaviors related to risk-taking (e.g., drug and alcohol abuse and sexual activity) were excluded because these behaviors were thought to involve different processes as related to MI. Participants were considered to be adolescent if they ranged in age from 12 to 18 years, which has been used in adolescent outcome studies by the World Health Organization [57]. Studies included in the systematic review were empirical papers, review articles, or case studies. Reviews and case studies were included because they were thought to offer insight and provide rich detail into the contexts and mechanisms through which MI works. Finally, studies had to be published in English. Relevant studies were identified through online searches of several relevant databases (i.e., PsychINFO, PubMed, Cochrane, and Healthstar). Search terms (with both American and British spelling) that were used to search the literature included (1) *motivational interviewing* or *motivational enhancement*; (2) *health behavior*, *diet*, *exercise*, *obesity*, *diabetes*, *sedentary behavior*, *nutrition*, OR *physical activity*; and (3) *adolescent*, *teen*, or *youth*. These terms were searched in the abstracts of studies. In addition, reference lists from review articles were checked for any other potentially eligible studies.

**Fig. 1:**
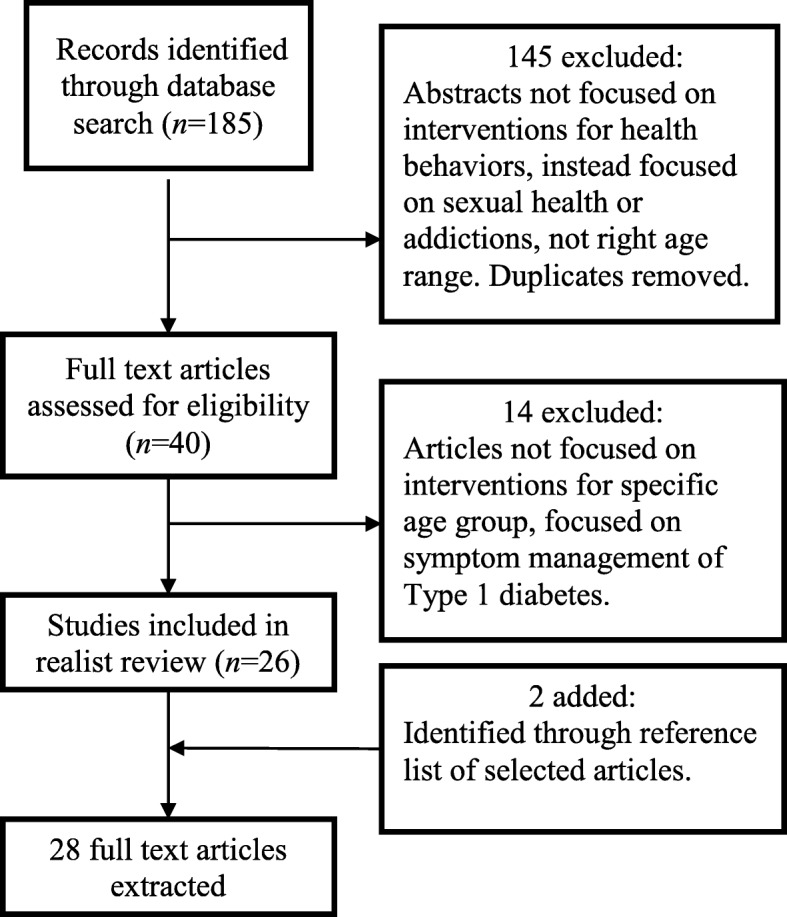
Flowchart of search strategy

This search strategy generated a total of 185 abstracts that subsequently underwent abstract review. Two researchers independently reviewed the list of abstracts and initial decisions were made on whether to retrieve the full text article. In the case of disagreement, discrepancies were resolved through discussion with a third research team member. Abstracts were excluded if they failed to meet one or more of the previously cited inclusion criteria. Selected (*n* = 40) abstracts were then retrieved and two members of the research team independently reviewed every article to confirm inclusion, abstract information, and complete a quality appraisal. Articles were removed from analysis primarily for the following reasons: lack of focus on MI, the majority of participants were not within the age range, and lack of findings or conclusions related to the health behaviors of interest. If MI was used with teens with diabetes, the article was only included if changing health behaviors was an outcome of the intervention. As a result, the full text for 26 articles were retrieved and coded for relevant information. Two additional studies were added from the reference lists of the coded studies (see Fig. 1).

### Appraisal of documents

Quality appraisal of articles was evaluated using the recommendations for realist review from RAMESES [58]. The rigor and relevance of the article was considered, rated as low, medium, or high. An appraisal deemed “low” was used for articles that did not include any information or discussion of mechanism or contextual factors. A rating of “medium” was given to studies that provide information on either contextual or mechanism variables. A rating of “high” was given to studies that provide information on both mechanism and contextual variables. Rigor was assessed as low, medium, or high, based on the credibility and trustworthiness of the method that was used in each article [58]. Studies appraised as low in rigor or relevance were excluded from the realist review. Two authors appraised each article; discrepancies were discussed and resolved in order to facilitate an iterative process of quality appraisal.

### Extraction and synthesis of findings

Once the articles that met inclusion criteria were identified, full text articles were retrieved and relevant information for analysis was extracted. Extracted information was related to four topics: (1) a description of the population(s) that was included in the study and other contextual factors of the study (e.g., healthcare setting); (2) a description of the program or intervention of interest (MI or the combination of MI with another intervention), (3) with the exception of review papers, a description of the evaluation was required; and (4) a description of the mechanisms or the underlying causal factors that might be contributing to the success or failure of the program.

The research team initially met and discussed preliminary mechanisms or theories (self-determination theory; competence, autonomy, relatedness). Once the candidate theory was clarified, it was tested and refined by examining articles that the research team had extracted. Iterative theory development was done across the papers and was consistent with the data analysis standards from RAMESES [58]. The focus on the data extraction was on contexts, mechanisms, and outcomes. Then, through an iterative discussion process, the theory was further developed by the creation of demi-regularities that included the creation of context-mechanism, context-outcome, or mechanism-outcome links. Lastly, thematic overlap from the demi-regularities was further analyzed in order to create the final CMO configurations.

In this qualitative process, an immersion/crystallization qualitative approach was used and researchers independently immersed themselves in the articles in order to crystallize, or decide on reportable themes or mechanisms [59]. Crystallization of the final contexts and mechanisms, as they related to MI for adolescent populations, was done through legitimizing and corroborating [59]. The current review was not registered in PROSPERO.

## Results

### Summary of studies

As noted in Fig. 1, there were a total of 28 articles included in this review and the most common study design included in this review was randomized control trials (*n* = 13). The next most common type of article was pre/post, correlational, or repeated measures design (i.e., seven in total). As well, two protocols, four reviews (including two book chapters), and two papers reporting secondary analyses (one qualitative) were included in this review. Most of the studies were of medium or high relevance (i.e., 23 in total). Health behaviors of target by all of the studies included were diet and exercise (see Table 1).

**Table 1:** Description of studies included in the realist review for MI and health behavior change for adolescents

Reference	Population characteristics	Description of MI intervention	Study design	Primary outcomes	Contextual factors	Mechanisms	CMO configuration
Ball et al. 2011 [74] (medium)	- 13 to 17 years old - BMI > 85th % - 46; 40% of these dropped out - Weight management clinic - Caucasian majority	- 16 to 20 weeks, 16 sessions total - MI and CBT delivered by RD and RN with 2 days of training - Parents invited to attend 3 parent-only sessions on supporting teenagers	- RCT; 2 intervention groups or wait-list control - Pre/post - No MI fidelity assessed	- %Δ of BMI *z* score; body weight; BMI and BMI percentile; improved in the intervention groups only - No difference with the addition of MI	- Caucasian majority - Clinical setting	- Lack of relatedness	- Lack of parental involvement (C_3a_) with Caucasian majority (C_4_), created lack of relatedness (M_3_), resulting in null results
Bean et al. 2015 [68] (medium)	- 11–18 years (M = 13.8) - African American (73%) females (74%) - Parent/caregiver willing to participate - MI (*n* = 58) or control (*n* = 41) - Attrition: 20.6%	- IG: Brief MI sessions on first and last session - Biweekly dietician and behavioral support visits and 3×/week supervised physical activity. - 2-day training by MINT trainer and 30 h practice. - Parental involvement but separate from MI	- RCT; pre, post follow-up at 3 and 6 months - High MI fidelity	- MI participants had greater 3-month adherence overall and to dietician and behavioral support visits, and result was consistent at 6 months	- Majority female, African American	- No mechanisms discussed	- High MI fidelity by clinician (C_2_), led to greater adherence to dietician and support visits - No mechanisms or behavioral change outcomes.
Berg-Smith et al. 1999 [73] (medium)	- Ages 13–17 - 50% female - Previously enrolled in DISC program for 3 years - *N* = 127 - MI session held in clinic - Reported no attrition	- Single session MI - Increase participant motivation + adherence to DISC dietary guidelines - Master’s degree level health educators and nutritionists. Interventionists had 18 h of training and ongoing supervision	- Pre/Post design - No control group - Follow-up 1–3 months - Did not report fidelity	- Mean proportion of calories from fat decreased from 27.7–25.6% - Proportion of calories from fat decreased from 9.5–8.6% of total energy intake	- Clinical setting - Family engaged for 3 years previous	- Reported that teens liked being treated as adults and wanted to express their own choices about what and how much to eat. - Relatedness within family	- Majority Caucasian (C_4_) Families already involved in intervention (C_3a_), created relatedness (M3) resulting in outcomes - Participants reported autonomy (M2), resulting in outcomes
Black et al. 2010 [60] (high)	- Aged 11–16 - 97% African Americans - Intervention completion and follow-up *n* = 91 and 89 - Control completion and follow-up *n* = 93 and 90	- Challenge program: mentorship + MI - 12 weekly sessions with mentor - Mentors received 40 h of training and had weekly supervision during the intervention	- RCT; pre/post design - Follow-up 24 months after end of intervention - Attrition 76.2% completed follow-up - Fidelity not assessed	- Overweight/obese status declined 5% among intervention adolescents and increased 11% in control - Increase physical activity - Decreased snack/dessert consumption	- Delivered in home and community - College-enrolled (or recently graduated) mentor - African American adolescents	- With a mentor, the adolescent experiences healthy eating and PA and gain confidence to adopt new behaviors	- African American adolescents (C_5_), MI delivered by gender- and race-matched peer (C_3b_), produced relatedness (M_3_), resulting in health behavior change
Brennan et al. 2008 [95]; Brennan et al., 2012 [79]; Brennan, 2016 (medium)	- Ages 11–18 and parents - 46% female - N: standard interview (SI) 34 - MI: 29 - 81% Australian - Mid to high income	- CHOOSE HEALTH program: MI + CBT or structured interview + CBT or wait list - MI session conducted with both adolescent and parent present	- RCT - Control group: yes - Attrition: reported none - No fidelity reported	- MI + CBT and SI + CBT did not differ significantly in terms of fat mass, lean mass and percent body fat, weight, BMI, BMI *z* score, waist circumference, waist–hip or waist–height ratio	- Parental engagement - At an Australian university - Delivered by a post-graduate in psychology - MI session was conducted with both the parent and adolescent present	- Report that the efficacy of MI in the current study may have been influenced by parental involvement in the session	- MI not delivered effectively (C_2_), adolescents could not develop autonomy (M_2_), leading to null findings
Carcone et al. 2013 [69] **(**medium)	- *N* = 40, with primary caregivers - Recruited from pediatric and endocrinology clinics - Participants were self-identified black adolescents - 27 females - M age 14.7 - Mean BMI was 38.5 - Low- to middle-class families	- Identify interventionist motivation patterns and language that are most successful - Counselors highly trained in MI by the MINT - Four 60-min sessions of MI - Change plan was completed and shared with the caregiver at the end of the session - The counselor met with the caregiver alone (20 min)	- Coded by SCOPE, adapted to capture culturally relevant examples of CT and CM - Analysis of codes	- 62% of the time, counselors’ open-ended questions elicited CT - Provider statements emphasizing autonomy were more likely to elicit CT - Affirming statements not effective	- Parental involvement - Highly trained professionals - Met with parent/child separately. - African American adolescents	- Provider statements emphasizing adolescents’ autonomy or personal choice in making health-related decisions were highly predictive of adolescent CT	- Highly trained professionals in MI (C_2_); provider statements asserting autonomy leads to adolescent change talk (M_2_). - No outcomes reported
Carcone, et al. 2016 [70] (medium)	- 37 adolescent/parents dyads - Self-identified as black - Secondary data analysis - M age was 14.7 (SD = 1.63) and most were female (*n* = 27)	- Participants received a single MI session, approximately 60 min long - Sessions conducted by MI counselors who were members of the MINT - Counselors met first with adolescents alone, then with caregivers alone and ended with both together	- Extracted phrases assigned the ambivalence code - A total of 268 statements were extracted from 25 (67.6%) families - Directed content analysis - Fidelity not assessed	- Ambivalence is reported at higher rates for caregivers than youth - Ambivalence is less for nutrition-related changes for caregivers compared to youth - Greater convergence in ambivalence for nutrition-related changes - Greater divergence in ambivalence for physical activity-related changes	- Parental involvement - African American adolescents	- Ambivalence between adolescent and parent	- Parental involvement (C_3a_), divergence in the ambivalence between the dyad (M_4_), no outcomes reported
Davis et al. 2011 [80] (medium)	- *N* = 45 - Female, (BMI) ≥ 85th percentile - Latino - Average age = 15.8	- Participants received circuit training (CT) exercise training 2 times per week for approximately 60–90 min per session for 16 weeks - Participants were required to attend at least 28 of the 32 sessions. - MI group received 4 individual MI and 4 group MI sessions - Interventionist members of MINT	- Randomized to 1 of 3 groups: control (*n* = 13), circuit training (*n* = 18), or CT + MI (*n* = 14) - Pre/post test - The average of code was 3.8, with 3.5 being considered proficient	- MI sessions did not significantly improve health outcomes, and CT alone showed more promising results	- Trained interventionist - MI sessions were too frequent (8 sessions for 4 months) and were held before or immediately after the exercise sessions - Latino adolescents	- Lack of autonomy due to requirement to attend classes - Perform specific goals that were not their own in the exercise training portion	- MI delivered without fidelity due to goals not being collaborative (C_2_), lack of autonomy (M_2_), no significant outcomes
Flattum et al. 2009 [78] (medium)	- Girls at risk for becoming overweight or who are overweight - 41 girls (age M = 17) - 20 participated in the MI condition - Majority white (*n* = 11) - Attrition, 81% completed all seven sessions	- New Moves: individual sessions with MI, teaching nutrition and social support - 5 in person (20–25 min) and 2 phone visits (10–15 min) every 2 to 3 weeks - Registered dietitian and health educator - 2-day training in MI and also attended weekly case mgmt	- Mix-methods: coaches completed process evaluation forms, about goals, barriers to meeting goals, and setting of an action plan - No fidelity data	- Set goals 100% of the time - Achieved goals 75% of the time - Goals related to physical activity, nutrition, and social support - No outcomes	- Delivered in community/school setting - 2-day training in MI and attended weekly case management - Delivered by a dietician and health educator - MI phone sessions difficult to schedule	- Goal setting	
Gourlan et al. 2013 [62] (medium)	- *N* **=** 54 (28 in Standard Weight Loss Program (SWLP); 26 in SWLP + MI) completed interventions - Attrition rate = 13% - Recruited from hospital by gen. practitioner due to obesity - M age = 13 years - 41% female - BMI over 90th age and gender-specific percentiles	- Participants randomly assigned to groups - SWLP group received 2 individual sessions of 30 min at the hospital with a healthcare provider discussing health behavior - MI condition = plus 6 MI phone sessions - Doctoral student delivered MI - MI training including 40 h of reading and 32 h of training with the French Association of MI	- RCT - MI measured using MITI coding below proficiency for 2/5 ratings (reflection-to-question ratio and percent MI adherent) - Administered at baseline, 3 months, 6 months	- No difference in BMI - Significant increase in physical activity for SWLP + MI group - No difference in intrinsic motivation, perceived competence - SWLP + MI condition perceived medical staff as more autonomy supportive	- Hospital setting - MI adherence	- MI group had a significant change in integrated (i.e., engaging in an activity because it is perceived as coherent with his/her values and identity) and identified regulations (i.e., engaging in an activity because it is perceived as personally important and useful)	- MI adherence (C_2_), adolescent perceived staff as more autonomy supportive (C_2_), developed autonomy, and led to increased health behaviors
Lee and Kim 2015 [63] (high)	- Male students from a junior high school in Seoul who had BMI greater than 25 kg/m2. (*n* = 125) - Average age = 15.37 - 89.7% completed	- ME sessions 2×/week reinforcement - 16 weeks total (5 days a week and a total of 80 sessions). - Behavior-based motivational enhancement intervention applied in this study was based on materials used in previous studies - Text message sent to participants and parents	- Pre-post design (8 weeks) - No control group - No info on training or fidelity of ME	- BMI decreased - Physical activity increased - Self-efficacy and perceived benefits of exercise increased - Perceived barriers decreased - Significant increases in weight control and “better outlook” - Physical satisfaction lack of competence and tiredness were significantly reduced	- Intervention conducted in the gym and classroom of a middle school before the school day began - All male participants	- Self-efficacy - Increased perceived benefits to weight loss - Increase in weight control, better outlook, and physical satisfaction - Decrease in perceived barriers - Reduced lack of competence and tiredness	- School setting (C_1_), led to increased self-efficacy, reduced competence (M_1_), resulting in health behavior change
Love-Osbourne et al. 2014 [96] (low)	- Adolescents with a BMI > 85% - 2 school-based health centers located in public schools - 87% in the CG and 77 students (94%) in the intervention group completed study	- Both groups received preventive services - IG had a mean of five visits with the educator (range, 1 to 8). - IG randomized to receive either weekly text messages or no text messages for the first semester - Full-day training on MI techniques conducted by a local expert and a follow-up session with the trainer 2 months later	- BMI, demographic questionnaire - Pre/post - Record weight weekly and lifestyle behaviors daily on a paper log sheet - Participants were instructed to turn in log sheets weekly - No MI fidelity assessed	- CG had more youth who decreased their BMI compared to the IG (40 versus 18%) - CG had higher sports participation than IG (47 versus 28%) - Increased visit number not associated with improved BMI outcome. - No difference for text messaging group	- Age of student impacted outcomes (younger than 15 years had better BMI outcomes) - Unequal sports participation rate in the control group	- No mechanisms reported	
Lydecker et al., 2015 [97] (medium)	- N/A	- N/A	- Review from book chapter	- Interventions based in community settings are more successful - School-based interventions allow the adolescent to feel more comfortable - Family interventions are successful to create common goals. - Community workers are more culturally inclined and aware of the environment	- Book chapter - Various contexts - Comparison between community and hospital settings	- Autonomy - Self-efficacy - Readiness and willingness to change	
Macdonnell et al., 2013 [82] (medium)	- *N* = 49 - Caregiver/adolescent dyads - Health clinic - 13–17 years of age - African American	- Control group—nutritional program - Intervention group—MI sessions - Four 60-min sessions - Met with adolescent first, then dyad together - Dietician underwent 16 h of training, received weekly supervision from a network of MI trainers	- Pre/post - No fidelity reported	- Only 27% of the intervention group and 36.4% of the control group received all sessions - Decrease in fast food consumption - IG showed increased intrinsic motivation for physical activity but a decrease in activity - No change in BMI, or motivation for nutrition change, or fruit and vegetable intake	- Hospital setting - Low engagement - Family participation - African American adolescents	- Increased intrinsic motivation for physical activity	- African American (C_5_), family participation (C_3a_), resulting in low participation, and few outcomes
Mehlenbeck & Wember, 2008 [66] (medium)	- Book chapter - Adolescents + parents	- MI as a major component of the studies reviewed	- Review chapter, so varied by study	- Increasing physical activity - Improving nutrition - Diabetes self-management	- Varied by study - Family influence must be considered when changing health behaviors - Role of family members needs to be addressed	- Increased self-efficacy for making changes - Support self-efficacy by enhancing personal responsibility and ability to carry out behavior change - Self-confidence in achieving goals	
Naar-King et al., 2016 [81] (medium)	- 12–16 years old. - 67% (*n* = 122) female; mean age was 13.75 years - African American - Youth and caregiver	- Dyad was randomized to 3 months of home-based versus office-based delivery of MI plus skills building - After 3 months, nonresponders were rerandomized to continued home-based skills or contingency management - Sessions to reduce food intake by 500 kcal or to consume a maximum of 1600–2000 kcal per day. - 80 h of MI training	- RCT - Measured at baseline, 3 and 7 months - After 3-month data collection, families were randomized based on response and nonresponse to phase 1 treatment - MI fidelity computed (not reported)	- Attendance of sessions higher for home-based group - Greater weight loss for youth with higher executive functioning (no group differences) - No difference for percent overweight between groups or across time - No differences between skills or contingency management programs	- Location of program delivery (home versus office) impacts attendance - Clients with higher executive functioning have greater weight loss (in short term but not long term) - African American	- Clients were not able to develop a sense of autonomy (M_2_) because the clinician set the goals (reduction by 500 kcal)	- Youth with better decision-making skills (M_5_) are more likely to lose weight in the short term
Nansel et al. 2015 [67] (medium)	- 136 parent-youth dyads (treatment *n* = 66, control *n* = 70) - Aged 8–16 (*m* = 12.8 ± 2.6) - 90% Caucasian, high income - Type 1 diabetes diagnosis ≥ 1 year - Outpatient diabetes center - Retention through study completion was 92% - All participant withdrawals were in the IG	- 9 in-clinic sessions delivered to the child and parent - Control condition comprised equivalent assessments and number of contacts - Research assistants who received training in motivational interviewing delivered the intervention	- RCT - Dietary intake was assessed using diet records at 6 time points - The Healthy Eating Index 2005 (HEI2005) and Whole Plant Food Density (WPFD) were used for diet quality - No MI fidelity assessed	- At 18 months, HEI2005 was 7.2 greater and WPFD was 0.5 greater in the intervention group versus control, during which time the intensity of the intervention had decreased - There was no difference between groups in HbA1c across the study duration	- Parental involvement - Children with type 1 diabetes - Caucasian, high-income families	- HEI2005 and WPFD demonstrated improvement from months 12–18, during which time and the intensity of the intervention had decreased. - Adolescents had the opportunity to use their autonomy	- Caucasian (C_4_), parental involvement (C_3a_) leads to relatedness in the dyad (M_3_) creating improved diet quality - Outcomes occurred when the intervention intensity decreased, when adolescents could use their autonomy (M_2_)
Neumark-Sztainer 2008 [98]; Neumark-Stainzer 2010 [64] (high)	- 100% female - Obese or at risk for becoming obese - Mean age 15.8 - More than 75% racial/ethnic minority - *N* = 182 (intervention) and 174 (control) - Advertised as an alternative to the required physical education class - Attrition 80.8% completed 5 to 8 MI sessions	- New Moves - Physical education class - Nutrition education, empowerment, + individual MI sessions - MI, 5 to 7 times per year, every 3 to 4 weeks for 15 to 20 min - New Moves coaches were intervention staff who received training and ongoing support in MI	- Group RCT design - Control group: yes (inactive treatment) - Pre/Post/follow-up - No fidelity assessed	- New Moves did not lead to significant changes in percentage body fat or BMI - Improvements for sedentary activity, eating patterns, unhealthy weight control behaviors, and body/self-image - Significant decrease in total sedentary activity	- School setting - Majority racial/ethnic minority - IG reported more support for physical activity from friends, teachers, and family members than control - For healthy eating, significant increases were found for friend and teacher support, but not for parent support	- Intervention increased stage of change for physical activity, physical activity goal-setting behaviors, their self-efficacy to overcome barriers to physical activity, and perceived athletic competence	- School setting (C_1_) supports competence (M_1_), leading to increased health behaviors - Ethnic minority (C_5_) and peer involvement (C_3b_) led to feeling more supported by those in their life (M_3_), resulting in outcomes
Olson et al. 2008 [59] (medium)	- *N* = 148 intervention and 136 TAU - Family medicine practice - Adolescents - 50% female - 96% Caucasian - Medicaid rates from 10 to 40% - Attrition: none reported	- Healthy teens = MI + personal digital assistant - 1 brief MI session - 3 h of interactive training in MI by psychologists	- Pre/6-month follow-up - Control group - Attrition: none reported - Used self-report to measure diet and exercise - No fidelity assessed	- Significant changes for milk intake and physical activity - Specific predictors of improvement in physical activity level after 6 months were the Healthy Teens intervention group and an interest in making a change at baseline	- Clinical practice - Delivered by clinicians - Fewer health risks than adolescents screened in schools	- Interest in changing behavior at baseline predicted outcomes (M5)	
Pakpour et al. 2015 [65] (high)	- Obese adolescents - Outpatient pediatric clinic in Qazvin, Iran - 357 Iranian adolescents (aged 14–18 years) - Approximately 50% female - 119 in each treatment group - 113, 118, and 115 completed the 12-month assessment	- Randomized into MI intervention or an MI intervention with parental involvement (MI + PI) or assessments only (passive control). - 2 trained interventionists delivered all sessions - 6 MI sessions with youth - Parents in MI + PI group (*n* = 119) received 1 MI session in clinic delivered at the end of the 6 sessions	- RCT; pre/post - All MI sessions were audiotaped and quality checked by (MITI) instrument. All scores were above proficiency except percent complex reflections, which was slightly below proficiency	- Significant differences in favor of the MI + PI intervention for BMI changes, diet, physical exercise, and self-efficacy for diet - The MI + PI group was not superior to control for servings of vegetables and milk products per day, waist circumference, or social functioning	- Parental involvement - Outpatient clinic - Proficient professionals	- The intervention targeted the adolescents (6 sessions), with only 1 session given to parents; promoted autonomy; and perceived competence of the adolescent	- Parental involvement (C_3a_) promoted relatedness between parent and adolescent (M_3_) and resulted in changes in outcomes - High MI fidelity (C_2_) led to changes in health behaviors
Resnicow et al. 2005 [35] (medium)	- *N* = 123 - Recruited from churches - Adolescent + parents - 100% female - Ages 12–16 - African American - Overweight or at risk for becoming overweight - 84% completion - 73% completed follow-up	- Go Girls = nutrition education + two-way pager + MI + parent outreach - High-intensity group: 4 to 6 MI phone calls - Parents met alone for half of the session and then joined daughters for the physical activity and food tasting - Master’s level counselors received 16 h of MI training plus ongoing supervision	- Group RCT - Pre/post design - Control group: yes (6 sessions of education) - No fidelity assessed; authors state that their MI protocol was not appropriate for adolescents	- Average attendance in the high-intensity group was 13 of 23 (57%) sessions. - In the high-intensity condition, an average of 4 of 6 MI calls were completed. - No significant group differences - Girls who attended more than 75% of sessions had lower percentage body fat and BMI than those who attended fewer	- Delivered in church - Delivered by a master’s- or doctoral-level psychologists - Delivered MI over the phone - Developmentally inappropriate protocol - African American girls	- Only 45% stated calls helped them think differently about health habits - 47% agreed their counselor asked too many questions - The protocol may not have been developmentally appropriate	- MI protocol was inappropriate (C_2_); girls reported calls were not helpful (M_2_), resulting in no outcomes
Walpole et al. 2013 [99] (medium)	- 40 (females = 23) participants - Recruited from Toronto East General Hospital—convenience sample - M age was 13.9 - *N* = 20 (treatment condition), *n* = 22 (control condition) - Majority Caucasian with 2-parent households - BMI in obese range at baseline	- Standard care program—Healthy Lifestyles, participants randomly assigned to receive this care combined with either MI or social skills training (control arm) - 6 therapy sessions over the course of 6 months, at the time of their regularly scheduled Healthy Lifestyles appointments - Clinical psychology doctoral student - Training was 60+ h with the MINT	- RCT - Pre/post test - Sessions coded using the MITI 3.0 scale - Fidelity assessed: - MI treatment scored 2.7 for evocation, 3.1 for collaborative, 3.2 for autonomy supportive, 3.4 for direction, 3.5 for empathy; with an average global score of 3.2	- No significant differences in self-efficacy and eating habits - Both groups improved	- Clinical psychology doctoral student - Hospital setting - MI interventions lacked fidelity - Therapies were meant to be structured differently - Both groups implicated tactics in similar ways	- No mechanisms discussed	- Intervention lacked fidelity (C_2_), resulting in no outcomes

### Outcomes

Nearly every study focused on achieving outcomes related to increasing physical activity and changing eating habits, with varying results between the interventions. Olson and colleagues [60] reported increased physical activity at 6-month follow-up after an MI intervention and increases in milk servings at a 6-month follow-up; however, no changes were reported for fruit intake, vegetable intake, and sweetened beverage consumption. Black and colleagues [61] reported that although there were no changes in daily activity counts for the group of adolescents, those who were overweight or obese at the start reported increases in daily activity counts, compared to those who were in the normal range for weight. They also found decreases in caloric intake in general, as well as reduced consumption of snacks/desserts, marginal significant decreases in dietary fat and fried food, and marginally significant increases in fruit intake (in addition to the aforementioned activity counts examination) [61]. Resnicow and colleagues [35] reported that when asked about the telephone-based MI intervention specifically, 47% of teens reported it had a lot of influence on their physical activity and 42% reported it had a little influence. Additionally, 47% of teens reported that the MI intervention phone calls had a “lot of influence” on their diet and 45% of teens reported that it had a “little influence.” Forty-five percent of teens reported that the intervention helped them think differently about their eating habits, but no significant differences between groups were found for diet- or physical activity-related outcomes. Gourlan and colleagues [62] implemented two individual sessions with a healthcare provider discussing health behavior. In addition, the experimental group also received the same intervention plus six additional MI sessions, which was found to lead to a significant increase in physical activity at 6 months. Lee and Kim [63] reported that male students from a junior high school in Seoul who received ME twice a week for 16 weeks had a significant increase in physical activity.

Neumark-Sztainer and colleagues [64] found improvement regarding portion control among teenage girls at a 9-month follow-up. The authors also found significant improvements for sedentary activity, eating patterns, unhealthy weight control behaviors, and body image [64]. Pakpour and colleagues [65] implemented MI as adolescent only, or with parental involvement. They found that the group receiving MI with parental involvement had significantly different outcomes including BMI changes, diet, physical exercise, and self-efficacy for diet. The MI with parental involvement was not superior to MI alone for servings of fruits, vegetables, and milk products; waist circumference; or psychosocial functioning.

Two documents examined physical activity and diet outcomes in the context of diabetes management [66, 67]. These studies approached the treatment of diabetes from a self-management perspective and focused on supporting youth to achieve physical activity and nutrition outcomes as a means to self-manage their diabetes. Nansel and colleagues [67] found that at 18 months, scores on the Healthy Eating Index 2005 were 7.2 greater and scores on the Whole Plant Food Density scale were 0.5 greater in the intervention group versus control. Notably, there was no difference between groups in HbA1c levels across the study duration.

A number of studies also examined secondary outcomes related to the MI intervention. A study by Bean and colleagues [68] found that MI participants had greater 3-month adherence to dietitian and behavioral support visits, which was consistent at 6 months. Carcone and colleagues [69] implemented a secondary analysis of coded MI sessions and found that 62% of the time, open-ended questions elicited change talk by the adolescent. Additionally, statements emphasizing autonomy were more likely to elicit change talk. These authors also found that affirming statements were not effective in eliciting change talk [69]. Carcone and colleagues [70] carried out a secondary analysis of MI sessions with youth and their caregivers. The results indicated that caregivers reported more ambivalence than youth overall, but caregivers had less ambivalence than youth for nutrition-related changes [70]. Conversely, youth had less ambivalence for activity-related changes compared to their caregivers. The authors reported the greatest divergence in ambivalence was between youth and caregivers for physical activity-related changes [70].

Very few studies examined readiness to change as outcome measures. Channon and colleagues [71, 72] found that the majority of patients (84%) reported changes towards the action stage at 12-month follow-up. Berg-Smith and colleagues [73] included participants who varied in their readiness to change at pre-intervention and found that readiness significantly increased by one point on a scale from one to 12; action plans were made by 94% and successfully implemented by 84% of participants. Neumark-Sztainer and colleagues [64] found that 13% more participants in the MI intervention group progressed from pre-contemplation to contemplation in comparison to the control group.

Although BMI and weight were typically included as an outcome measure in studies, there was little evidence to support the impact of MI or MI in combination with other techniques at reducing BMI [62, 74, 75]. One exception is research by Black and colleagues [61] who found the percentage of obese/overweight adolescents in their control group increased in weight by 11%, yet the percentage decreased by 5% in the intervention group. Irby and colleagues [76] noted an improvement in BMI; however, the study’s quality and rigor are weak because it is a case study. Additionally, Lee and Kim [63] found a significant reduction in BMI when MI was used in addition to an obesity intervention. The results of this study should also be interpreted with caution as no control group was used for comparison [63]. A reduction in BMI may not be considered an achievable outcome given that adolescence is a period of relative weight gain and growth and efforts to counteract this would be futile. Furthermore, BMI may not be an ideal outcome because adolescents undergo a number of developmental physical changes that can impact BMI scores [77].

### Context—mechanism configurations

#### Structure—competence configuration

**Fig. 2:**
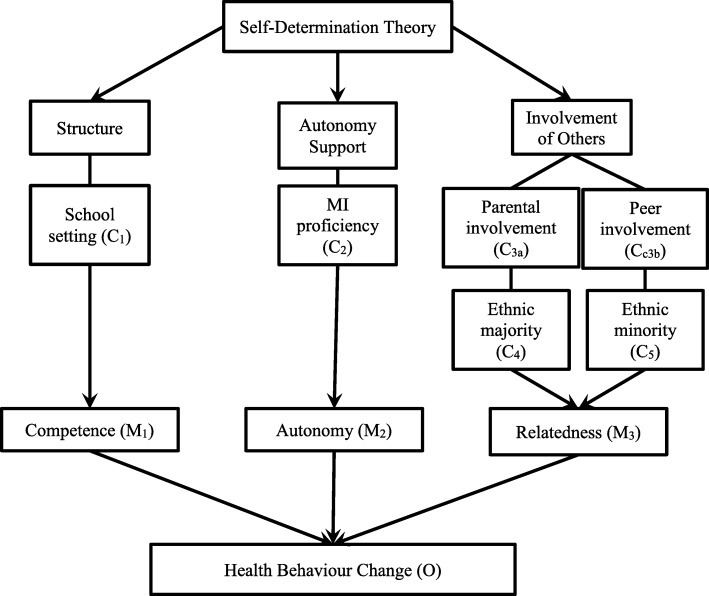
Realist theory of MI for adolescent health behavior change

The first context from the theory involved the therapist providing structure for the client, which in turn leads to the development of competence (see Fig. 2). The context of structure was found in the current review to occur when MI is delivered in a school setting (C_1_), providing structure that is familiar to the adolescent. Flattum and colleagues [78] as well as Neumark-Stainzer and colleagues [64], employed the New Moves intervention for ethnically diverse girls in a school setting. These studies found that due to the structure provided by the school setting (C_1_), girls increased their perceived athletic competence (M_1_) and their self-efficacy to overcome barriers for physical activity (M_1_). In this study, changes in girls’ levels of competence were paralleled with changes in health behaviors. Lee and Kim [63] provided ME to male students with a BMI over 25 kg/m^2^ twice per week for 16 weeks in a gym and classroom before the school day began. Due to the structure of the school context, the students were found to have increased self-efficacy, decreased perceived barriers, and increased competence (M_1_), leading to health behavior changes. Various studies were conducted using MI in other contexts including health clinics, home based, and over the phone. These contexts were found to have mixed results and therefore cannot be used as a definitive contextual variable.

#### Autonomy support—autonomy configuration

The second context from self-determination theory is autonomy support, where the individual making a change must be supported in their autonomous decision-making and not be pressured to act in a specific way. This context creates the behavior change mechanism of autonomy (M_2_). For the present review, autonomy support was found when interventionists had high fidelity to MI and were delivering MI in a way that respected the autonomy of the client (C_2_). Carcone and colleagues [70] performed a secondary analysis of MI sessions conducted by highly trained counselors. The sessions were coded by the Sequential Code for Observing Process Exchanges (SCOPE) in order to identify what elicits change talk for adolescents. The authors found that provider statements that emphasized the adolescent’s autonomy (C_2_) were more likely to elicit change talk (M_2_). Additionally, they found that statements that were affirming were the least effective. These results suggest that when MI is provided in a way that supports the autonomy of the client, the individual will be more likely to feel autonomy over their own choices, leading to more discussion of behavior change. Gourlan and colleagues [62] measured fidelity to MI using the Motivational Interviewing Treatment Integrity (MITI) coding proficiency and found ratings above proficiency for three out of five ratings. These authors found that adolescents who received the MI intervention perceived staff as more autonomy supportive than those who did not receive MI (C_2_). Additionally, those in the MI group had a significant change in engaging in an activity because it is consistent with their own values and because it is personally important and useful (M_2_). These results are consistent with what has been previously been identified as autonomous motivation, which arises from the autonomy-supportive approach of MI.

#### MI non-adherence

A number of studies reported null results that were explained by the way MI was delivered. For example, Brennan and colleagues [75] found that one session of MI followed by 12 sessions of CBT did not improve outcomes compared to those who only received CBT. They found that among the treatment group, there was only a 43% completion rate. Interestingly, in a follow-up paper by these authors, they discuss the barriers that led to treatment dropout in this study. They reported that the most common barriers reported by adolescents related to research demands, such as completing questionnaires, as well as the treatment approach, program components, and strategies, which were discussed as having too many behavior change goals and too much homework [79]. These results suggest that the CBT program following the MI session did not support adolescents’ autonomy, which is likely to have contrasted immensely from the initial MI session, leading to ambivalence for the adolescent to change their behaviors. Additionally, the most common barriers reported by parents were the same as adolescents, including program components and strategies such as too many behavior change goals, too much homework, and adolescent dislike of monitoring [79]. Therefore, it appears that this program did not provide the context of autonomy support for adolescents, leading to lack of mechanisms and outcomes.

Davis and colleagues [80] provided Latino females circuit training twice per week for 16 weeks, in addition to four individual and four group MI sessions. The MI sessions were found to have high fidelity, with the average MITI code being 3.8, but circuit training alone was found to show more promising results. One explanation is that the youth were expected to perform specific goals during the exercise training portion that were not their own. Additionally, participants were required to attend 28 of the 32 sessions and could not miss more than two consecutive sessions. Therefore, it appears that the circuit training differed greatly from the MI spirit, which could be the reason for the null results for the MI intervention.

Reniscow et al. [35] recruited female adolescents from churches and conducted MI with the adolescent through phone calls. This study found no significant difference between those who received MI and those who did not. Interestingly, the authors state that although the MI protocol was successfully implemented in adult studies, the counselors believed the protocol might not have been developmentally appropriate for the adolescents. Results indicated a lack of MI fidelity and autonomy support, including that 47% of girls agreed their counselor asked too many questions.

Lastly, Naar-King et al. [81] delivered an MI intervention to youth and their parents either in their homes or in an office setting. After 3 months of the intervention, treatment non-responders were re-randomized to receive skills training or contingency management. The results found no differences between the sessions delivered in home-based or office-based settings and no difference between skills training or contingency management. This intervention does not adhere to the MI spirit, as individuals who were not ready to make a change were randomized into another behavioral condition. Additionally, during the MI sessions, the goal was to have participants to reduce their food intake by 500 kcal or to consume a maximum of 1600–2000 kcal per day. Therefore, adolescents did not have the opportunity to set their own goals, which contrasts greatly to the MI approach. The results of the above studies show that it is necessary to employ an intervention that follows MI fidelity guidelines, including all parts of the intervention, not only the MI itself.

#### Involvement of others—relatedness configuration

The final context from self-determination theory is the involvement of others (C_3_), which produces the mechanism of relatedness (M_3_). This context was found to occur in two different ways: the involvement of parents (C_3a_) and the involvement of peer mentors (C_3b_). These two contexts were found to have different outcomes depending on the other contexts of the adolescents. Specifically, it was found that parental involvement is more likely to lead to positive outcomes for Caucasian, middle- to high-income families (C_4_). For ethnic minority families (C_5_), the involvement of peers (C_3b_) was more likely to lead to positive outcomes.

With respect to family involvement, Nansel and colleagues [67] delivered MI to youth and their parents at a diabetes outpatient center. Participants were 90% white, high-income families, with a study completion rate of 92%. Due to their high levels of treatment engagement (C_4_), adolescent healthy eating increased during the 12–18-month period, at which time the intensity of the intervention had decreased and the adolescents had the opportunity to use their autonomy (M_2_). Ball and colleagues [74] delivered individual MI to a majority Caucasian sample and found no differences between the MI intervention and the control. Interestingly, this intervention targeting the adolescent alone had a 40% dropout rate, and the authors cited a lack of family engagement as a reason for this high dropout rate. Therefore, in order for MI to be effective in ethnic majority groups, it is necessary that parents be also targeted in the intervention. These results parallel Nansel et al. [67] and Berg-Smith et al. [73] suggesting that parental involvement (C_3_) may be important for developing the mechanism of relatedness (M_3_) for Caucasian families (C_5_).

An RCT conducted by Pakpour et al. [65] in an outpatient clinic in Iran involved MI with the addition of parental involvement at the end of every session. There was a high degree of involvement by participants indicated by the low dropout rate (C_3a_). This study indicated that the MI + parental involvement resulted in significant changes in health behavior change outcomes. In contrast to the above studies, MacDonell et al. [82] conducted MI with African American adolescents and their parents. Only 27% of the intervention group received all MI sessions, which suggests that there may be a difficulty in engaging with the parent-adolescent dyad in this intervention, but due to the limited data, this lack of engagement is a provisional result. The results of this study did find a reduction in fast food consumption and an increased motivation for physical activity, but no other significant outcomes were presented.

With respect to peer involvement, Neumark-Stainzer et al. [64] elicited the context of involving others by creating an intervention for female, ethnic minority youth within a school setting. This intervention involved sending postcards home to reinforce New Moves with parents (C_3a_) and having informal lunches with the group (C_3b_). The results of the involvement of others in this intervention led to the intervention group reporting more support for physical activity from friends, teachers, and family (M_3_). Additionally, the MI group also reported increased support from friends and teachers for nutrition-related changes (M_3_). Black and colleagues [61] created the Challenge program, which involved MI delivered by race- and gender-matched mentors (C_3b_). The authors found that through the interaction with the mentor (M_3_) in experiencing physical activity and nutrition, the adolescent gained confidence to adopt new behaviors (M_1_), which led to healthy behavior change. Both Neumark-Stainzer et al. [64] and Black et al. [61] suggest the importance of peer involvement among youth who are an ethnic or racial minority. Interestingly, interventions with ethnic minority participants that did not include peer involvement were found to have no intervention effects [35, 73, 76].

### Insufficiently explored contexts and mechanisms

A small number of studies mentioned contexts and mechanisms that were not found to fit within the SDT framework. For example, Olsen and colleagues [60] found that an interest in changing behavior at baseline was a significant predictor of outcomes at follow-up. Additionally, a study by Carcone and colleagues [70] of African American adolescents and their caregivers compared the frequency of ambivalence statements during an MI session with the dyad. The results found that overall ambivalence was higher for caregivers than for youth. The ambivalence between the dyad was similar for nutrition-related changes, but diverged for physical activity-related changes. Parents were more likely to be ambivalent towards their adolescent making physical activity-related changes compared to youth. The role of ambivalence within the parent-adolescent dyad may be an important variable to further explore in order to better understand how the ethnic minority context and the mechanism of relatedness is activated in this population.

Flattum and colleagues [78] monitored adolescent girls’ goal-setting behaviors and found that when receiving MI, girls were able to set goals 100% of the time and achieved goals 75% of the time. Neumark-Sztainer and colleagues [64] paralleled this result and found that the MI intervention increased physical activity goal-setting behaviors of adolescent females. The role of goal setting as a mechanism of change for health behaviors is still unknown. Lastly, Naar-King et al. [81] found that individuals with higher executive functioning had greater weight loss in the short term, but not the long term.

## Discussion

The purpose of this realist review was to explore the role of self-determination theory in the implementation of MI for adolescent health behaviors, with the goal of ultimately providing useful guidance for program development and implementation. Previous research has indicated that self-determination theory can be used to explain the efficacy of MI for behavior change [53]. The mechanisms responsible for initiating health behavior change originate from self-determination theory: competence, autonomy, and relatedness. School-based programs were found to bring about the mechanism of competence, leading to behavior change outcomes. Secondly, interventionists who were proficient at MI were found to initiate the mechanism, autonomy. The contexts of family engagement and ethnic majority were found to trigger the mechanism of relatedness. Conversely, the contexts of peer involvement and ethnic minority triggered the mechanism of relatedness.

The CMO configuration of structure-competence was found to occur for youth within a school setting. Previous research has indicated that the school setting is essential in providing youth with activities that are challenging but that also allow for sufficient support and feedback, in order to promote competence and success [83]. The school-based programs in the present review were consistent with other educational goals, allowing for competence in health behavior change to develop. Research in educational contexts has shown the importance of setting limits with students in order to obtain the structure necessary to learn. Koestner et al. [84] found that autonomy-supportive limits result in significantly higher intrinsic motivation in comparison to controlled limits. Therefore, the present review has indicated that the MI intervention benefits from being delivered in a setting with sufficient structure (e.g., school setting) that also is consistent with the autonomy-supporting spirit of MI, including having the adolescents set their own goals.

Relatedness was found to be a key mechanism for adolescents to change their health behaviors. Research has indicated that people tend to internalize the values of others who they want to feel connected to in order to feel a sense of belonging [83]. The present review found that this mechanism was triggered by two different contextual variables: family involvement and peer involvement. Having members of the family or peers who the adolescent wants to feel similar to was found to be an important component to successful health behavior change. Future interventions would benefit from involving family and peers in the intervention to successfully target this mechanism.

The context of ethnic majority and minority in relation to the mechanism of relatedness is a demi-regularity that must be further explored in future realist reviews. A meta-analysis reviewing 25 years of studies on MI found that studies including a higher number of African American adult patients had significantly worse outcomes [85]. These results were only significant when MI was compared to treatment as usual. The authors state that it is unclear why the number of African Americans is a significant moderator, whereas the number of Hispanic Americans was not significant. It appears that utilizing MI with those who identify as an ethnic minority may result in varying outcomes. Importantly, this meta-analysis did not report on any specific details related to the age groups of participants receiving MI, the setting in which the MI was delivered, nor whether who delivers the MI to ethnic minority groups has an effect on outcomes [85]. The present review provides an interesting insight into these difficult issues by suggesting that for ethnic minorities, MI needs to be delivered to adolescents with support from peers, rather than parents.

Shavers and colleagues conducted a review of research on racial and ethnic discrimination in the healthcare system [86]. Results of their analysis indicated that African Americans experienced the highest amount of ethnic-based discrimination, ranging between 6.9 and 52%. Discrimination was found to have negative effects including worse health status, non-adherence to treatment, mistrust of providers, and avoidance of the healthcare system [86]. Therefore, it is possible that African American parents may be less likely to engage with their adolescent’s healthcare due to previous discrimination by the healthcare system. It may be more helpful for relatedness to be achieved for adolescents through peers or ethnicity-matched providers.

It is apparent that MI with parental involvement is not *entirely* ineffective for different ethnic groups, as Pakpour et al. [65] employed a successful MI intervention with parental involvement in Iran which documented many positive outcomes. It may be that those from an ethnic minority may find it difficult to integrate an MI approach when the MI treatment provider is from a different ethnic group or socioeconomic status, especially if they have experienced previous discrimination by the healthcare system. Therefore, the best approach may be to “match” clients with a provider who is similar to them in order for relatedness to be achieved.

A recent realist review describes a population of hard-to-reach or marginalized families, describing this population as those in poverty or from a cultural minority [87]. These authors noted the difficulty to engage this population due to socioeconomic needs as well as matching the client’s lived experience. Socioeconomic status may also explain why parental involvement may not be effective for ethnic minority youth, as parents may not have the ability to join adolescents during their appointments or effectively support them, but this needs to be further explored [87]. The same review found that the context of matching the client’s lived experience was important for those who were from cultural minority groups, paralleling the CMO configuration, ethnic minority-relatedness-health behavior change, in the present study. More research is necessary to further unpack why peer mentors are more effective than family engagement for minority youth.

The present review has indicated that autonomy support from SDT is best characterized by fidelity to the MI spirit, which underscores respect for autonomy. This context was found to elicit the mechanism of autonomy for adolescents. Previous research in health behavior change has found that when patients perceive their health care providers to be supportive of their autonomy, patients are more likely to feel autonomy over their health behaviors [88]. This suggests that the more supportive autonomy providers are, the more patients perceive they have autonomy. This study provides further support for the demi-regularity identified in the present review. In addition, Williams et al., [88] also found that changes in patient autonomy leads to health behavior change, hypothesizing a complete CMO configuration that is also found in the present review.

A recent review analyzing the mechanisms of change within MI interventions found that the MI spirit is associated with positive health behavior change outcomes [89]. Interestingly, a number of articles in the present review did not report the fidelity of their MI intervention. Because MI adherence from the interventionist was found to be a contextual factor leading to autonomy, it is necessary for future research to document MI adherence. It was also found that many interventions contrasted with the basic elements of MI, such as the interventionist setting the goals for the adolescent, having a fixed number of sessions the adolescent has to attend, or having the adolescent participant engage in activities they do not want to. These non-autonomy supportive interventions were found to have no effect on behavioral change outcomes. A meta-analysis of 25 years of MI research has stated that adherence to a manual may cause interference with the client-centered approach of MI [85]. However, another meta-analysis reviewing MI for weight loss found that the use of a fidelity measure is associated with better outcomes [90]. Therefore, although a manual may not be the most appropriate answer for MI fidelity, it is necessary to adhere to the MI spirit, allowing clients to exhibit their autonomy during the process, without being rigid to specific goals or manuals. The present review, paralleled by previous research, suggests that adherence to a manual is not the only way to ensure high levels of fidelity. Importantly, being flexible and client-centered means adjusting according to the client, which is a guiding principle of the MI spirit.

A number of mechanisms were mentioned within articles, but were not extensively documented. These included readiness to change, executive functioning, and goal-setting behaviors. It is hypothesized that these mechanisms are related to both competence and autonomy. Miller and Rose [91] discuss the mechanism of change talk as central to health behavior change. More recently, Deci and Ryan [92] have argued that it may not be the quantity of change talk, but rather the quality, and that the provider must support the client in autonomous change talk rather than forcing a larger quantity of change talk. These results are similar to the insufficiently explored mechanisms in the present study. The results found that motivation to change and goal-setting behaviors are mechanisms of change. Previous research has highlighted the importance of goal setting and motivation to change in behavior across different age groups. Greaves and colleagues [93] conducted a systematic review of reviews in order to assess the intervention components associated with positive dietary and physical activity outcomes. Results found that behavior change techniques, including goal setting, were associated with increased intervention effectiveness. Additionally, Knittle and colleagues [94] provided a review and meta-analysis of interventions targeting motivation for physical activity in adults. This review also found that interventions that focus on goal-setting behaviors lead to increased physical activity. Interestingly, the review did not find a significant relationship between motivation and change in physical activity for adults, which parallels the present review for adolescents [94]. It is apparent that this mechanism needs to be further explored within behavioral change interventions.

In line with Deci and Ryan [92], motivation to change and goal-setting behaviors may not be able to occur unless the adolescent is able to autonomously move into a higher stage of change or make goal-setting behaviors on their own. The present review has indicated that if a provider is setting these goals for them, the adolescent will not develop competence or autonomy. Due to the lack of current understanding, the role of readiness to change, motivation, and goal-setting behaviors, and their relationship to autonomy and competence, needs to be further explored. It may be of interest to test the TTM and the mechanisms involved in the precontemplation and contemplation stages to further address the mechanisms not extensively documented in the present review.

### Limitations of review

A number of limitations exist within the present review. Importantly, this realist review did not have differentiated pathways for specific outcomes. The majority of studies looked at a combination of diet, exercise, and weight loss outcomes making it impossible to separate CMO configurations out by outcome. Rather, this review focused on contexts and mechanisms that lead to health behavior change generally. More research is needed in order to identify the pathways that lead to each specific health behavior change.

Second, a number of contextual factors were mentioned in articles, with no observable patterns of outcomes. For example, a number of interventions took place within a health clinic, in a community setting, or via telephone. It is possible future research can examine these contextual factors in order to assess their efficacy and create different CMO configurations. For the purpose of this review, it was concluded that not enough evidence currently exists to understand the impact of those contextual factors.

Due to the nature of realist reviews, some material needs to be omitted in order to focus the theory building in a rich and cohesive manner. Therefore, other aspects of the data can become neglected and unexplored, which are described as insufficiently explored mechanisms and contexts. It should be noted that future syntheses might choose to focus on different contexts and mechanisms within MI or to use a comparison theory in order to build additional CMO configurations.

## Conclusions

This original review is the first to provide critical information on the processes and contextual variables that impact the efficacy of MI for adolescent health behaviors. The realist-informed systematic review allowed for the inclusion of a variety of articles in order to analyze and create a clear theoretical understanding that can be utilized by future interventionists. Based on SDT, this review brings to light the importance of structure, autonomy support, and relatedness for adolescent health behavior change. Due to the provisional nature of the review, further exploration of mechanism variables not extensively documented such as motivation to change and goal-setting behaviors would improve our understanding of MI and be consistent with the present realist approach.

## Abbreviations

BMI: Body mass index
CBT: Cognitive-behavioral therapy
CMO: Context-mechanism-outcome
MI: Motivational interviewing
MITI: Motivational Interviewing Treatment Integrity
SCOPE: The Sequential Code for Observing Process Exchanges (SCOPE) coding
SDT: Self-determination theory
TTM: Transtheoretical Model
